# Climate Change Impacts on One Health Systems: A Narrative Review of Empirical Evidence and Policy Responses

**DOI:** 10.1002/puh2.70351

**Published:** 2026-08-18

**Authors:** Ioannis Adamopoulos, Aida Vafae Eslahi, Niki Syrou, Aikaterini Frantzana, Panagiotis Tsirkas, George Mpourazanis, Fredrick Kayusi, Konstantina Diamanti, Harshit Mishra, Maad M. Mijwil, Guma Ali, Elma Hrustemović, Raúl Argüello‐García, Demetris Lamnisos

**Affiliations:** ^1^ Department of Public Health and Policies, School of Social Science Hellenic Open University Pátra Greece; ^2^ Department of Public Health Policy Sector of Occupational and Environmental Health School of Public Health University of West Attica Athens Greece; ^3^ Medical Microbiology Research Center Qazvin University of Medical Sciences Qazvin Iran; ^4^ Department of Physical Education and Sport Science University of Thessaly, Karies Trikala Greece; ^5^ Department of Health Sciences European University Cyprus Nicosia Cyprus; ^6^ Department of Gynecological Surgery Hatzikosta General District Hospital Ioannina Greece; ^7^ Department of Obstetrics and Gynecology General Hospital “G. Hatzikosta” Ioannina Ioannina Greece; ^8^ Department of Environmental Studies Geography and Planning Maasai Mara University Narok Kenya; ^9^ Department of Environmental and Earth Sciences Pwani University Kilifi Kenya; ^10^ Department of Early Childhood Education and Care School of Social Sciences University of Ioannina Ioannina Greece; ^11^ Department of Agricultural Economics College of Agriculture Acharya Narendra Deva University of Agriculture and Technology Ayodhya India; ^12^ Digital Economy Department College of Administration and Economics Al‐Iraqia University Baghdad Iraq; ^13^ Department of Computer and Information Science Faculty of Technoscience Muni University Arua Uganda; ^14^ Department of Genetics and Bioengineering Faculty of Engineering, Natural and Medical Sciences International Burch University Sarajevo Bosnia and Herzegovina; ^15^ Department of Food Safety and Environmental Protection, Faculty of Veterinary Medicine University of Sarajevo Sarajevo Bosnia and Herzegovina; ^16^ Department of Genetics and Molecular Biology Center for Research and Advanced Studies (Cinvestav) National Polytechnic Institute (IPN) Mexico City Mexico

**Keywords:** climate change, global health security, health systems, One Health, policy responses

## Abstract

**Background:**

Climate change poses critical threats to human, animal, and environmental health, yet evidence on integrated One Health responses remains fragmented. Although numerous studies highlight climate‐driven health risks, many climate adaptation frameworks still underrepresent cross‐sectoral interactions and the needs of low‐resource settings.

**Objective:**

This study aims to identify and operationalize key governance mechanisms, surveillance strategies, and collaborative approaches that can be implemented to support health‐adaptive policies.

**Methods:**

A comprehensive search of peer‐reviewed literature and international reports (March 2020–June 2025) was conducted across Scopus, PubMed, Web of Science, the World Health Organization (WHO), WHO African region (AFRO), European Centre for Disease Prevention and Control (ECDC), the Food and Agriculture Organization (FAO), and the European Public Health Association (EUPHA) repositories. A narrative synthesis approach was adopted to integrate findings across heterogeneous study designs and contexts.

**Results:**

Climate change intensifies vector‐borne diseases, heat‐related illness, food insecurity, biodiversity loss, and environmental degradation, disproportionately affecting low‐ and middle‐income countries. Evidence suggests that implementing a One Health approach through effective, evidence‐based actions such as fostering collaborative engagement, using a common operating picture for community‐based surveillance, and strengthening resilient food and livestock production systems that are adaptable to a changing climate could enhance surveillance capacity and accelerate preparedness and early warning processes.

**Conclusion:**

Significant opportunities exist to improve One Health governance by, among other things, establishing coordinated surveillance systems, flexible policy approaches, an ecosystem‐based perspective, and inter‐sectoral engagement. Currently, policies fail to fully consider environmental and animal health aspects, which could pose significant risks.

## Graphical abstract

Schematic representation of the interaction framework for climate justice and One Health adaptation policies: The conceptual design illustrates the systemic barriers in public health and disaster medicine, mapping the core triggers and effects of climate change alongside key global challenges.

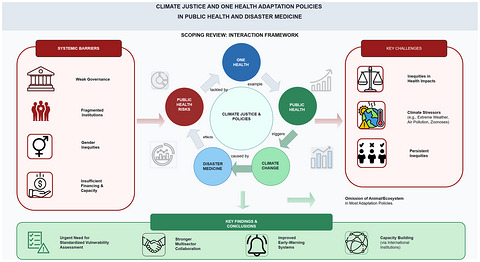

## 1 Background

Climate change is a significant global issue that threatens the One Health and livelihoods of a large population [1, 2]. The One Health Summit emphasizes the need to take a collaborative approach to address this crisis, focusing on preventive measures and scientifically informed strategies [3]. Climate change intensifies existing health challenges and introduces new challenges [4], compounded by population growth, urbanization, and human encroachment on wildlife habitats [5]. This increases the risk of health impacts, particularly in lower income countries, affecting food security, safety, nutrition, and overall health [6]. Climate change also hinders progress in addressing poverty, conflict resolution [7], gender discrimination, healthcare access, and other critical components of the sustainable development agenda [8, 9]. A holistic approach is crucial for comprehensively assessing the current situation and the quality of the available evidence, identifying relevant risk factors and mitigation strategies, and defining achievable outcome targets [10]. One Health approach to adapting to climate change can play a crucial role in enhancing food security by addressing various aspects, such as food and nutrition security, environmental sanitation, and climate change responses across multiple species, to mitigate biodiversity loss [11]. The interconnectedness of health has gained support through the “One World–One Health” initiative [12]. However, it is important to note that current discussions regarding the health impacts of climate change have focused primarily on human health, often neglecting indicators related to animal health [13, 14]. The One Health approach emphasizes the shared nature of health among humans, animals, and the environment and underscores the need for sustainable improvements in social and environmental services both locally and globally [15].

To address the objectives of this study's climate change impacts and policy responses to the One Health approach, cooperation, consistent procedures, and a commitment to addressing intricate new health risks are all needed, according to the gaps in the literature. To identify and assess essential policy instruments for creative policies based on technical, environmental, organizational, and regulatory developments influencing climate change and one's health, strategic pathways for the evolution of intelligent, human‐centered, and flexible policies should be suggested. Recent analyses of healthcare systems emphasize the importance of organizational resilience and supply chain adaptability in addressing complex health challenges [16, 17].

The aim and scope of this study are to investigate new opportunities in climate change impacts on One Health and policy responses and to provide a roadmap for how governance, global policies, organizations, and regulatory bodies may adjust to an increasingly complex risk environment in One Health. The study also aims to assess the current readiness of health systems to respond to climate‐related and digital risks and to investigate models for intelligent policymaking and decentralized risk response. The key target areas include One Health systems, climate‐adaptive risk planning, human‐centered design, and the worldwide harmonization of One Health and public health standards.

## 2 Methods

### 2.1 Search Strategy and Study Selection and Data Extraction

Narrative synthesis protocol for this review was developed on the basis of existing guidelines on conducting and reporting of narrative synthesis of evidence in health research. As the evidence on climate change and One Health is very broad and heterogeneous, the narrative review was developed to map the landscape of the evidence, synthesize findings across a range of study designs, and identify the gaps in governance at a policy level. A wide search in databases, including Scopus, PubMed, Web of Science, and databases and reports from international organizations like the World Health Organization (WHO), WHO African region (AFRO), European Centre for Disease Prevention and Control (ECDC), Food and Agriculture Organization (FAO), and European Public Health Association (EUPHA), was conducted to gather papers and reports between January 2020 and June 2025.

The search string included “climate change,” “global warming,” “climate variability,” “extreme weather,” “heat waves,” “One Health,” “human–animal–environment interface,” “public health,” “policy response,” and “health systems.” These terms were combined using AND and/or other Boolean operators. Literature search was carried out on 15/06/2025. Following screening of titles, abstracts, and full texts for relevance, suitable studies, consisting of systematic reviews, meta‐analyses, and policy in‐depth analyses, were included for this narrative review. The selection process included systematic reviews, meta‐analyses, and comprehensive policy analyses that synthesized evidence on climate‐sensitive health outcomes, assessed policy interventions, and included integrated approaches to One Health strategies. The literature was analyzed to find topics and issues that require integration for health, with a focus on benefit of greater human–animal collaboration when dealing with climate change‐related challenges. As the body of evidence regarding climate change and One Health is heterogeneous, qualitative synthesis of findings of the conducted systematic reviews, meta‐analyses, and high‐quality narrative reviews was included. This was to map the terrain of evidence and reveal the gaps in policy‐related governance.

### 2.2 Inclusion and Exclusion Criteria

A search for climate change and One Health relevant articles was screened, resulting in adjectival publications. Publications were categorized to health impacts of climate change, response systems for managing the threats to human health caused by climate change, and integrated approaches in the context of such policy responses.

Selected studies included only systematic reviews, meta‐analyses, and extensive narrative reviews of existing evidence on the climate change impacts upon the One Health systems and their policy responses. Individual studies were excluded from the scope of this review as it was intended to highlight global patterns in evidence and identify gaps in governance and recommendations for future policies. In addition, it was chosen to adopt a narrative review design, which aims to create a global overview in line with global policy decision‐making.

The search criteria pertaining to the paradigm framework were followed, with one of the resulting categories addressing One Health impact, risk management responses, and prospects of integrative responses in policy responses, strategy, and program management.

### 2.3 Narrative Synthesis Approach

**FIGURE 1:**
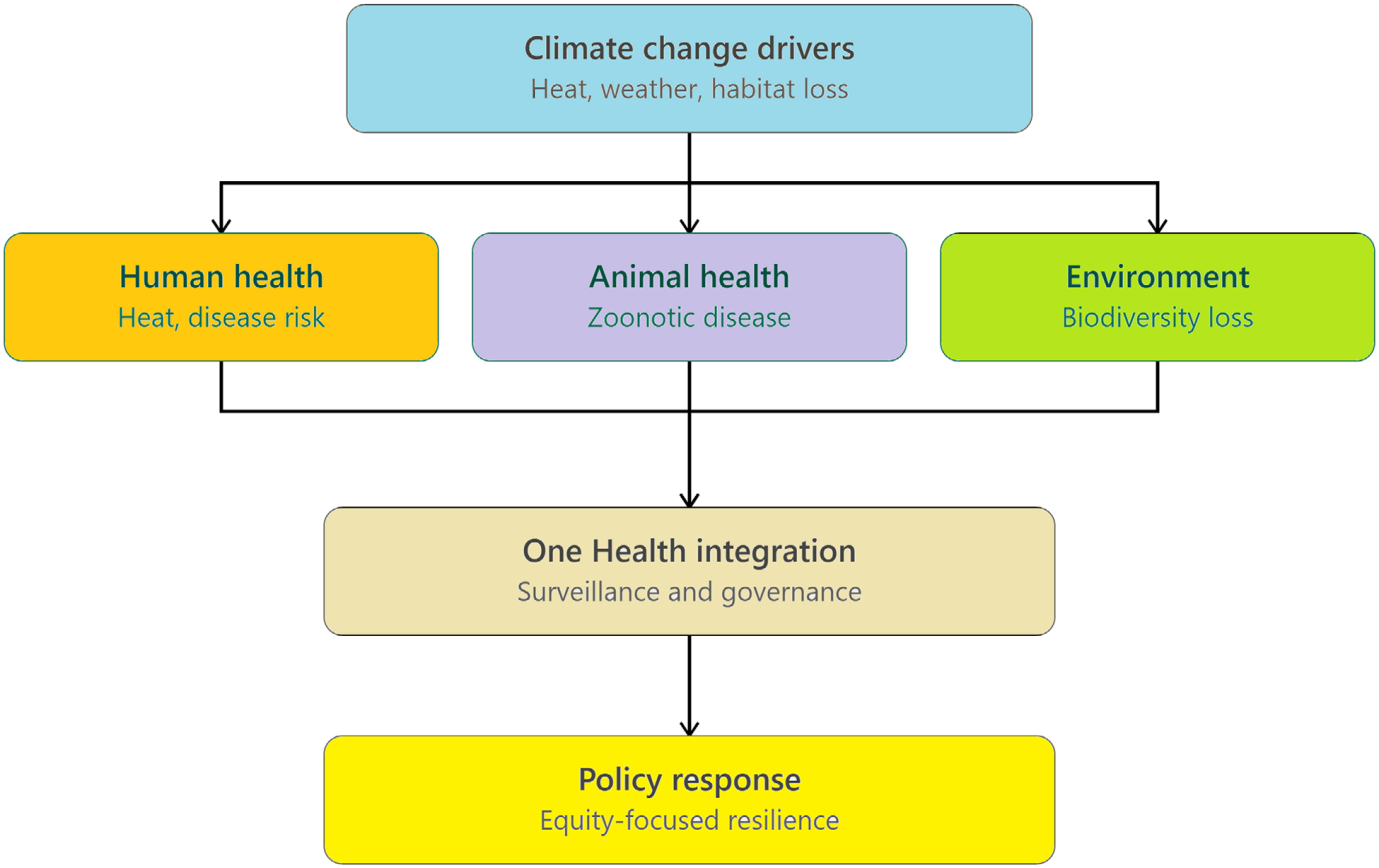
Conceptual framework illustrating the cascading pathways from climate change drivers to One Health outcomes and policy integration.

The thematic synthesis of this narrative review was informed by the conceptual framework (Figure 1) and organized according to five thematic domains: climate drivers, human health impacts, animal health impacts, environmental change, and equity‐focused policy responses. Because the articles included in this review vary widely in terms of scope, study design, and results, a narrative synthesis approach was undertaken. The key elements of this approach include (1) organizing articles according to the thematic areas described above, as well as policy responses and policy and governance gaps, (2) describing themes and variation across studies, (3) describing the evidence base for each domain, and (4) synthesizing findings and drawing an overall interpretation.

### 2.4 Methodological Constraints

The long‐term impacts of climate change on One Health are limited by methodological issues, preventing us to have a complete insight into the net of interacting processes. To understand heat waves and their contribution to explosive outbreaks of parasites is only possible through a large multi‐county study with rich time series on meteorological parameters and a high resolution on space–time epidemiology time series. Many datasets over time on parasites, vectors, and reservoir hosts needed for long‐term modeling are not sufficiently resolved or missing at all. Although many new early‐warning systems for societal and environmental emergencies and prediction are coming along, there are still scientific problems on decoupling climate change and variability to the megatrends of changing global.

## 3 Results

### 3.1 Study Selection

A large volume of peer‐reviewed literature and grey literature and organizational reports was identified in the literature search. Once filtered for relevancy against the specific research question pertaining to the climate change impacts upon the One Health system and the policy interventions in response, a select group of robust evidence sources was included and subject to an in‐depth thematic synthesis. The evidence sources selected consisted of systematic review papers, meta‐analysis, comparative policy studies, and major international policy reports from key stakeholders, such as the WHO, AFRO, FAO, ECDC, and EUPHA.

### 3.2 Key Findings Across Studies

#### 3.2.1 Impacts of Climate Change on One Health

Reports were repeatedly showing higher numbers of vector‐borne diseases, increases in heat‐related illnesses, a rising tide of flood‐related health effects, and impacts to biodiversity in relation to climate stressors. Populations most at risk from environmental exposures in countries with low‐ and middle‐income are also those with low adaptation capacities. Climate change is also considered a major health and environmental inequity issue, with far more negative effects on health and greater health costs than on food productivity or infrastructure damage [18]. The burden of climate‐sensitive diseases is greatest for the poorest populations, highlighting health inequity [19]. Many of the health impacts of climate are particularly threats to poor people in low‐ and middle‐income countries (LMICs) [20]. The risk of mortality from weather‐related natural disasters is 50 times greater, the health costs related to flooding are more than 200 times greater [21], and the risk of mortality from vector‐borne diseases is almost 300 times greater in developing nations than in developed countries [22]. Urban settings create local climate conditions that cause most direct human health hazards due to the urban‐heat‐island effect, increased air pollution, flooding, or water shortages [23].

The health sector is engaged almost nowhere in climate change mitigation and adaptation planning, which is crucial to address the increasing number of deaths and diseases related to pollution from the burning of fossil fuels, increasing incidence, and spatial expansion [24]. The intensity of extreme weather events and the development of knowledge and skills at the local level are needed to implement prevention/adaptation programs [25]. To address these threats, actions need to be taken in the health system to strengthen primary healthcare and develop preventive programs [26].

The emergence or re‐emergence of infectious and vector‐borne diseases associated with changes in climate, human habitation, and agricultural practices [27, 28]. Global warming, a phenomenon affecting the Earth's temperature, has led to significant social, economic, and environmental changes [29]. The Earth has warmed by 2°C over a century, causing catastrophic secondary changes that become irreversible at 3°C [30]. This simultaneous increase in temperature and other changes significantly impacts vulnerable regions [31].

The spectrum of climate‐driven hazards extends across multiple transmission pathways, ranging from direct heat exposure to complex vector‐borne and water‐borne dynamics. These pathways, along with their corresponding evidence‐based interventions, are systematically summarized in Table 1, which links specific climatic stressors to concrete health outcomes and recommended adaptive strategies.

**TABLE 1:** Climate drivers, specific health threats, and evidence‐based intervention pathways.

Climate driver	Specific health threat	Transmission/Pathway	Recommended intervention strategy (evidence‐based)
Rising temperatures/Global warming	Expansion of vector‐borne diseases (e.g., dengue, malaria, zoonotic arboviruses)	Vector–human spillover; shortened pathogen incubation periods	Integrated community‐based vector surveillance; early detection in livestock reservoirs before human cases occur
Extreme weather events (flooding, storms)	Water‐borne disease outbreaks (e.g., cholera), flood‐related injuries, and mental health impacts	Contaminated water sources; physical trauma; displacement	Strengthen primary healthcare resilience; establish early‐warning response systems; climate‐proof water sanitation infrastructure
Drought/Heat waves	Heat‐related morbidity/mortality; food insecurity; livestock productivity loss	Crop failure; heat stress on animals; reduced dietary diversity	Climate‐adaptive livestock systems; diversify agricultural supply chains; implement heat‐health action plans
Habitat disruption/Biodiversity loss	Emergence/Re‐emergence of zoonotic pathogens (e.g., Nipah, Ebola); wildlife–livestock–human interface risks	Human encroachment on wildlife habitats; disrupted ecological balances	Ecosystem‐based interventions; integrated syndromic surveillance across wildlife, domestic animals, and humans; habitat conservation linked to health governance

#### 3.2.2 Environmental and Animal Health Dimensions

Climate variability disrupts livestock systems, contributes to emerging zoonotic pathogens, and reduces food security. Multiple studies highlight insufficient integration of veterinary surveillance, ecosystem monitoring, and environmental indicators into health policies.

Globally, human, animal, and plant health are being significantly impacted by climate variability and extremes [32]. Storms, droughts, floods, and heat waves are some of these consequences [33]. However, many global frameworks ignore the wider range of environmental conditions necessary for the survival of both untamed and domesticated species of plants and animals in favor of concentrating on infrastructure, agriculture, and human health [34]. Efforts to lessen the adverse effects of climate change on people, animals, plants [35], and ecosystems are hampered by inadequate awareness and governance of environmental elements, which leads to incorrect responses from health systems [36]. Health is a complex issue that is intertwined with environmental vulnerabilities [9, 36]. A health approach that integrates health into ecosystems can help address the impact of climate change and its effects on health [37]. One Health approach emphasizes animal‐source foods, extensive livestock systems, adequate water, and environmental sanitation [38]. It significantly contributes to food security and health equity [39]. This approach can also help reduce health risks associated with climate change [40]. A robust and transparent implementation of this approach can significantly contribute to food security and health equity [41].

#### 3.2.3 Policy and Governance Responses

There is evidence to suggest that effective multi‐sectoral and inter‐sectoral collaboration enhances disease outbreak preparedness and contributes to overall cost‐effectiveness in healthcare systems. Integrated, community‐based surveillance systems have played a vital role in the timely identification of new health hazards. There are obstacles to good governance in relation to these issues, including uncoordinated policies, underinvestment, inadequate information, and a lack of inter‐sectoral cooperation. The health method or One Health method is aimed at providing better integrated answers to connected well‐being problems in opposition to a range of disciplinary borders [42]. This approach involves transdisciplinary processes to design, implement, and evaluate integrated solutions for various dimensions, including societal, cultural, political, and economic aspects [43]. The solutions are often context‐specific, requiring broad application [44]. With climate change influencing health challenges, interest in integrated approaches and inter‐sectoral collaboration is increasing [45]. Solutions derived from scientific evidence at a broader governance level may be needed to address global health challenges [46, 47].

A comprehensive synthesis of these governance challenges, alongside the specific health impacts and proposed inter‐sectoral interventions, is provided in Table 2.

**TABLE 2:** Summary of climate change impacts on One Health domains with policy implications.

One Health domain	Key climate impacts	Health consequences	Policy gaps identified	Recommended policy responses
Human health	Heat waves, extreme weather, flooding, air pollution	Vector‐borne diseases, heat‐related morbidity, flood injuries, respiratory illness	Health sector rarely engaged in climate adaptation planning	Strengthen primary healthcare; integrate climate‐health into national adaptation plans
Animal health	Temperature shifts, habitat disruption, livestock stress	Emerging zoonotic pathogens, livestock disease outbreaks, reduced productivity	Veterinary surveillance underfunded and under‐integrated	Expand veterinary surveillance; integrate animal health into One Health frameworks
Environmental health	Biodiversity loss, ecosystem degradation, water scarcity	Food insecurity, malnutrition, waterborne diseases, habitat loss	Environmental indicators neglected in health policies	Implement ecosystem‐based interventions; monitor biodiversity and water quality
Food security	Crop failure, livestock stress, supply chain disruption	Malnutrition, foodborne illness, reduced dietary diversity	Agricultural and health policies poorly coordinated	Climate‐adaptive food systems; support smallholder farmers; strengthen food safety
LMICs and equity	Disproportionate exposure, weak adaptive capacity	Higher mortality, economic losses, deepened health inequities	Equity considerations under‐integrated in One Health frameworks	Equity‐centered adaptation; invest in low‐resource settings; community‐based surveillance

Abbreviation: LMICs, low‐ and middle‐income countries.

#### 3.2.4 Identified Gaps

**FIGURE 2:**
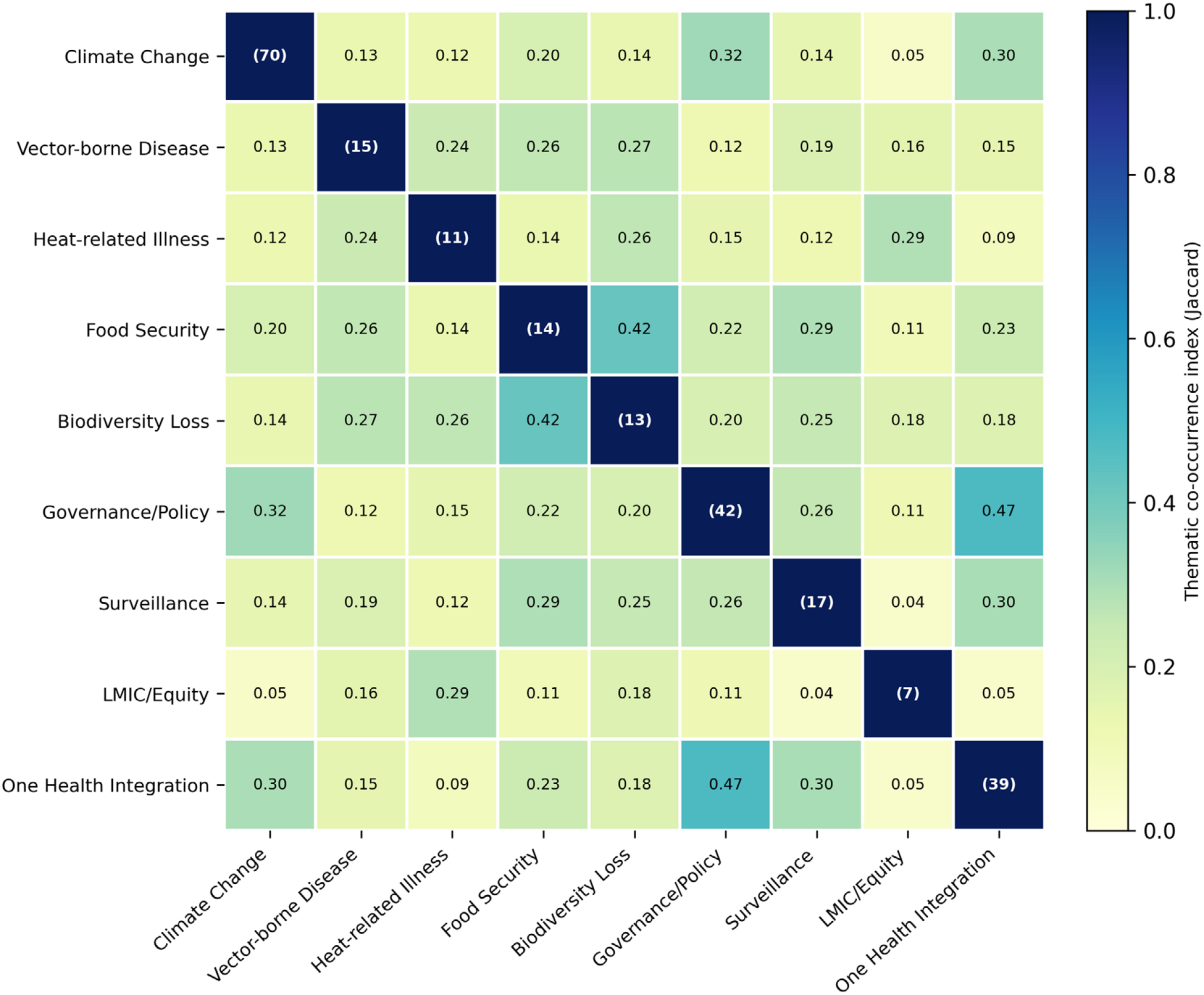
Thematic association heatmap of key One Health domains identified in the reviewed literature (2020–2025). Values represent normalized association strengths based on co‐occurrence and thematic clustering in included studies. Higher values indicate stronger thematic linkages (range: 0–1).

Key gaps include the lack of long‐term datasets for climatehealth modeling, insufficient consideration of animal and environmental health in human‐centered climate policies, limited implementation of existing adaptation measures in low‐resource settings, and inadequate collaboration among public health, veterinary, and environmental authorities. Figure 2 is generated with Python (version 3.12.3) and represents a thematic connection heatmap illustrating the links between major One Health sectors according to the literature. Several clear links appear to exist in the literature between governance/policy and the overall One Health integration (0.47), and between food security and the biodiversity loss (0.42). Links with LMICs/equity remained low in relation to other topics, which reflect the fact that equity remains difficult to integrate in operational ways with the existing concepts in the health‐related areas (0–0.2 on average).

### 3.3 Overall Synthesis

The One Health approach proves promising for combating climate‐related risks with an ability to strengthen an integrated surveillance approach, the monitoring of environmental and livestock systems, nature‐based solutions, and coordinated policies. Yet, the implementation of One Health is currently hindered by constraints relating to funding, governance, and poor intergovernmental policy coherence at regional and country levels.

### 3.4 Key Findings

Global change encompasses shifts in climate, environmental conditions, and demographic patterns, all of which can have substantial implications for human, animal, and ecosystem health. These changes may alter 1) the life cycles and dynamics of vectorhostpathogen interactions; 2) the seasonality and patterns of vectorhost transmission; 3) the prevention and control of diseases such as paratuberculosis, foot‐and‐mouth disease, and brucellosis; and 4) environmental health through the emergence and spread of wildlife diseases, the introduction and expansion of invasive alien species, and habitat loss. Addressing these interconnected challenges requires a holistic One Health framework that recognizes the interdependence of human, animal, and environmental health and supports coordinated strategies to mitigate the impacts of global change.

## 4 Discussion

The One Health approach to climate change focuses on addressing interconnected issues across sectors, including the health of interdependent biota and socioeconomic and political systems [48]. This approach emphasizes the importance of modeling biological and socioeconomic system behaviors and a preemptive response capacity to early‐warning signals [49]. One Health approach to climate change can significantly contribute to food security by emphasizing animal‐source foods, environmental sanitation [50], and integrated syndromic surveillance and response systems [51]. Case studies from urban, peri‐urban, and rural areas highlight the need for surveillance and early response of livestock and human syndromes likely driven by climate change [52, 53, 54]. This approach highlights the potential for co‐distributed policy interventions in the context of sociopolitical settings.

Climate change is a global concern, and countries are urged to design national action plans to meet global climate goals [55]. The health, agriculture, and livestock sectors must demonstrate their national action plans to mitigate and adapt to climate change [56]. The One Health system has been the focus of several global policies, including the FAO efforts to mitigate the impacts of climate change, environmental pollution, and the disruption of biological interaction chains [57]. The WHO/AFRO coordinated the establishment of a regional working group on animal–human health interface to combat zoonotic diseases and address the effects of climate change on the One Health system [58]. Global policies, such as the WHO's climate change and health coping strategies, focus on the human health side [59]. Addressing transboundary and emerging diseases of livestock; modeling links between climate change and human infectious diseases, animal and zoonotic diseases of wildlife, domestic reservoirs and vectors, biodiversity loss, and anthropogenic land use change [60]. However, WHO policy recognition of links between climate change and health, particularly food safety, is acknowledged [61].

Europe is implementing national multispectral One Health strategies to address climate‐induced risks for human, animal, and ecosystem health [62]. Governments are promoting collaboration platforms and objectives to mitigate risks [63]. Baseline assessments of health and climate risks are being conducted to highlight and prioritize risks for One Health and collaboration across sectors [64]. Health adaptation measures are being identified and implemented, with long‐term objectives being concrete and specific enough for immediate action [65]. Countries with similar climate risks, such as heat in Europe and insect‐borne diseases in Europe and South Africa, are jointly assessing risks and adaptation measures [66]. A variety of tools can assist in implementing multispectral plans, with a focus on infectious disease transmission, environmental exposure [67], health services, vector control, and health co‐benefits [68]. Monitoring and evaluation frameworks should be developed to enable continuous utilization of these tools and address unanticipated effects [69]. The global climate crisis has significantly affected human health, with AI‐driven analysis highlighting the negative effects on public health [70, 71]. This crisis has led to increased risks of workplace hazards among global populations, especially the public health workforce [72, 73]. The effects of heat stress on occupational safety, health, and hygiene are also evident [73, 74]. The future of the international food supply faces significant challenges, including climate change, environmental degradation, and population growth, highlighting the need for innovative policies and AI‐powered solutions. At the same time, air pollution and poor indoor air quality may disproportionately affect high‐risk and vulnerable populations, further exacerbating existing health inequalities. The findings and quality assessment of the included studies highlight the need for integrated strategies that address these interconnected environmental, technological, and health challenges [75, 76].

The evidence points to an increase in vector‐borne zoonotic pathogens due to climate change in low‐ and middle‐income countries. Veterinary surveillance is crucial to mitigate these risks, but the cost may be lower if the virus is detected early. Community‐based surveillance, involving participatory mapping and incentivized recordkeeping, can be a promising approach. This system supports literate norm‐entrepreneurs and informal veterinary service provisions along trade routes, empowering nanoscale entrepreneurship against human conflict and climate change. This non‐hierarchical, fragmentation‐tolerant organizational form is effective against human conflict and climate change. The health concept emphasizes sustainability in response to climate change. Understanding climate interactions and trends is crucial for optimizing adaptive strategies. The One Health concept, which includes mechanisms for food security, antimicrobial resistance control, and environmental sanitation, can help address these challenges and ensure sustainable health systems. Experience with other emerging diseases suggests that outbreaks of emerging vector‐borne zoonotic pathogens are much less expensive to control when they are detected early in the vector or livestock than when they are detected later at the human level. The integrated surveillance and response of a consortium of human and veterinary health authorities may be a promising avenue to reduce the health effects of climate change. Many trends remain theoretical or based on preliminary research, and regional inequalities in resources and technology may also cause delays in the adoption of the One Health approach to climate change, which focuses on addressing policy responses.

The exposure of health impacts in policy documents helps understand the prioritization of anticipated health risks. Climate change impacts, grouped by risk category, health effect, exposure sector, and world region, account for most of the 50 health impacts. Water availability, high‐temperature exposure, and disease vector spread pose more anticipated health risks than other categories do. Policy documents have focused mainly on high‐income countries and developing regions, with health impacts covering wider world regions being included in fewer documents. Countries with fair human development are less aware of the impacts of climate change on health than countries with high human development. Policymakers should ensure that sustainable, clean energy solutions are scaled up while managing health risks arising from energy transition strategies on the basis of research evidence. More research on the impacts of climate change on health is needed to better inform policymakers, especially in developing and emerging countries.

### 4.1 Limitations and Future Research Directions

The evidence base for this narrative review is characterized by considerable heterogeneity in terms of study design, scope, geographical focus, and methodological quality. Although this heterogeneity reflects the transdisciplinary and emergent nature of One Health research, it limits our ability to draw definitive conclusions and underscores the need for more standardized approaches to evidence generation and policy evaluation.

Second, although it is widely recognized that LMICs are most at risk from climate change, the evidence base reviewed in our study remained dominated by research conducted in high‐income countries, with relatively few reviews focusing specifically on LMIC contexts or providing extensive evidence from these settings. This imbalance limits the ability to generalize findings to the vast climate‐vulnerable regions of the globe.

Third, most of the included studies were based on cross‐sectional data or shorter term monitoring of climate and health‐related impacts. Longitudinal data to model interactions between climate and health outcomes, particularly those related to the animal and environmental sectors, are scarce. Consequently, it remains challenging to establish causal relationships and develop predictive models for early warning of climate hazards.

Fourth, the conclusions of this review rely heavily on the completeness, rigor, and transparency of the underlying systematic reviews and meta‐analyses. It is possible that these source reviews contain errors, biases, or limitations that could affect our findings. Additionally, primary studies published in languages other than English may not have been captured.

Fifth, although we searched institutional repositories of international organizations, such as WHO, FAO, ECDC, and EUPHA, we likely missed a large volume of local, national, and sub‐national policy documents that could provide valuable evidence on implemented policies in practice or the local conditions that facilitate or hinder policy implementation. Such documents have diverse formats and may vary greatly in quality and level of detail compared to published scientific literature.

Finally, the included reviews are mostly focused on presenting potential health risks and developing policies rather than evaluating policy outcomes, impact, feasibility, and sustainability. As a result, the available evidence does not present a clear picture of what policies have effectively delivered in terms of actual implementation.

## 5 Conclusion

Our study has found that our current policies designed for climate change on the whole are failing to result in appropriate adaptation across the board in our increasingly globally integrated world. Three questions were explored to this effect: (1) the effects of climate change in our current system; (2) the effectiveness of our current policy frameworks; and (3) effective adaptation measures. The data suggest that these are evident that climate change increases the number of vector‐borne diseases, a tendency toward food insecurity, an increase in heat‐related illnesses, and causes damage to the biodiversity on our planet.

These issues particularly affect lower and middle‐income countries significantly.

Currently, our policies do not integrate environmental and animal health indicators, thus creating problems with disjointed policy frameworks and inhibiting early action to contain problems. However, there is potential and hope, as integrated community‐based surveillance, multi‐sectoral collaboration, adaptive food and livestock systems, as well as operationalizing One Health have shown successful adaptations. The main findings consist of (1) harmonized multi‐sector surveillance leads to early intervention and is cost‐effective through community‐based approaches; (2) poor policy cohesion and a general avoidance of cross‐sectoral policies result in our inability to properly adapt, a problem exacerbated by lack of accountability for poor implementation; (3) funding long‐term data and research infrastructure enables more informed decision‐making for adaptive measures to be considered; and (4) adaptation mechanisms need to consider equity‐centered adaptation for all citizens, including those living in less affluent regions. Finally, our policy approaches to climate change need to incorporate more than just human health‐centered concepts. This is crucial as there are major gaps and potential for growth within current systems.

The current results of our study offer practical guidance for numerous groups. As policymakers should endeavor to form multiministerial One Health committees and mandate better data sharing, they must also aim to integrate the core elements of One Health within any existing climate adaptations. As practitioner groups, they should look at more holistic community‐based surveillance strategies, including their decision‐making regarding planning based on risk assessments.

International bodies are called upon to invest in prevention rather than reactive solutions and bolster support for veterinary and environment‐based data systems, particularly in those areas most at risk of the impacts of climate change.

Lastly, researchers must look into policy‐engaged implementation‐based research, aiming to increase general understanding of issues surrounding One Health policy in more accessible language and with standardizable metrics for all of their research into adapting to climate change. As, despite the limitations that this review faced, it clearly showed that it is no longer justifiable to delay action regarding this crucial component of human security simply because “further evidence” is lacking. The present time is now, a time for action to begin implementation and learning rather than contemplation in the vain hope of attaining perfect evidence. Translating the evidence shown in this review into comprehensive strategies focused on the principle of equitable adaptation is paramount in creating systems that enable resilience to climate change.

There are undoubtedly measures that we as humans are capable of making to mitigate the worst effects of our contribution to climate change on global One Health systems, but they require investment in many areas from surveillance and cooperation to the integration of systems, in addition to ongoing research and support for our communities globally.

## Author Contributions

**Ioannis Adamopoulos**: conceptualization, data curation, formal analysis, investigation, methodology, project administration, software, supervision, writing – original draft preparation, writing – review and editing. **Aida Vafae Eslahi**: methodology, data curation, supervision, writing – original draft preparation, writing – review and editing. **Niki Syrou**: investigation, methodology, writing – original draft preparation, writing – review and editing. **Aikaterini Frantzana**: investigation, methodology, writing – original draft preparation, writing – review and editing. **Panagiotis Tsirkas**: investigation, writing – original draft preparation, writing – review and editing. **George Mpourazanis**: investigation, writing – original draft preparation, writing – review and editing. **Fredrick Kayusi**: writing – original draft preparation, writing – review and editing. **Konstantina Diamanti**: writing – original draft preparation, writing – review and editing. **Harshit Mishra**: writing – original draft preparation, writing – review and editing. **Maad M. Mijwil**: writing – original draft preparation, writing – review and editing. **Guma Ali**: writing – original draft preparation, writing – review and editing. **Elma Hrustemović**: writing – original draft preparation, writing – review and editing. **Raúl Argüello‐García**: writing – original draft preparation, writing – review and editing. **Demetris Lamnisos**: writing – original draft preparation, writing – review and editing.

## Funding

The authors have nothing to report.

## Ethics Statement

This study is based solely on literature reviews and reports.

## Conflicts of Interest

The authors declare no conflicts of interest.

## Data Availability

All data used in this study are available from the corresponding author upon request.
